# Use of Kaolin-Impregnated Gauze Aids in Hemostasis and Blood Loss Mitigation in a Penetrating Injury to the Bladder and Small Bowel

**DOI:** 10.7759/cureus.46583

**Published:** 2023-10-06

**Authors:** Brandi Campbell, Christine Castater, Randi N Smith, Jason D Sciaretta, Jonathan Nguyen

**Affiliations:** 1 Surgery, Morehouse School of Medicine, Atlanta, USA; 2 Surgery, Emory University School of Medicine, Atlanta, USA

**Keywords:** trauma laparotomy, quikclot, penetrating injury, hemorrhage, hemostasis, kaolin impregnated gauze

## Abstract

Hemorrhage control can be technically challenging in penetrating injuries to the pelvis. In an era of decreased availability of blood, rapid hemostasis is critical to minimize blood loss, limit transfusions, and control contamination from hollow viscus injuries. QuikClot Control+® 12x12 Hemostatic Device(C+) (Teleflex Medical OEM, Plymouth, MN), a form of kaolin-impregnated gauze, maybe a helpful adjunct to ebb the flow of hemorrhage from large surface area wounds. We present a case in which C+ was utilized in the preperitoneal packing of a gunshot wound to the pelvis and aided in obtaining hemostasis while simultaneously allowing the team time to complete the remainder of the case. Though further large randomized control trials are required to identify the role of C+ in trauma laparotomy, it remains a tool in the surgeon’s armamentarium when dealing with hemorrhage.

## Introduction

A gunshot wound (GSW) may result in massive tissue destruction, and the subsequent cavitation can amplify the injury to surrounding vascular structures, soft tissues, and organs. This can cause a massive hemorrhage and exacerbate the patient’s hemodynamic instability by inducing acidosis, coagulopathy, and shock [1]. Hemorrhage from a GSW to the pelvis can often prove exceedingly difficult to control due to the potential for significant arterial and venous bleeding in a narrow operative field. The surgeon must focus on rapid hemostasis, resuscitating the patient, and control of contamination in a timely manner. In the current context of a critical national blood shortage declared by the American Red Cross, the former tasks can be especially difficult [2].

Hemostatic dressings like kaolin-impregnated gauze may have some benefits, as kaolin aids in the coagulation pathway to promote clot formation. Furthermore, the newest version of kaolin-impregnated gauze, QuikClot Control+® 12x12 Hemostatic Device (C+) (Teleflex Medical OEM, Plymouth, MN), offers substantial benefits over its predecessors, such as Combat Gauze® and TraumaPad®. For instance, the C+ mechanism of action allows it to be three times more effective than previous iterations, with less concern for the systemic absorption of kaolin from the gauze (unpublished data). We present a case of successfully using C+ as an adjunct for hemostatic control in a patient with a through-and-through GSW to the bladder and bowel.

## Case presentation

**Figure 1:**
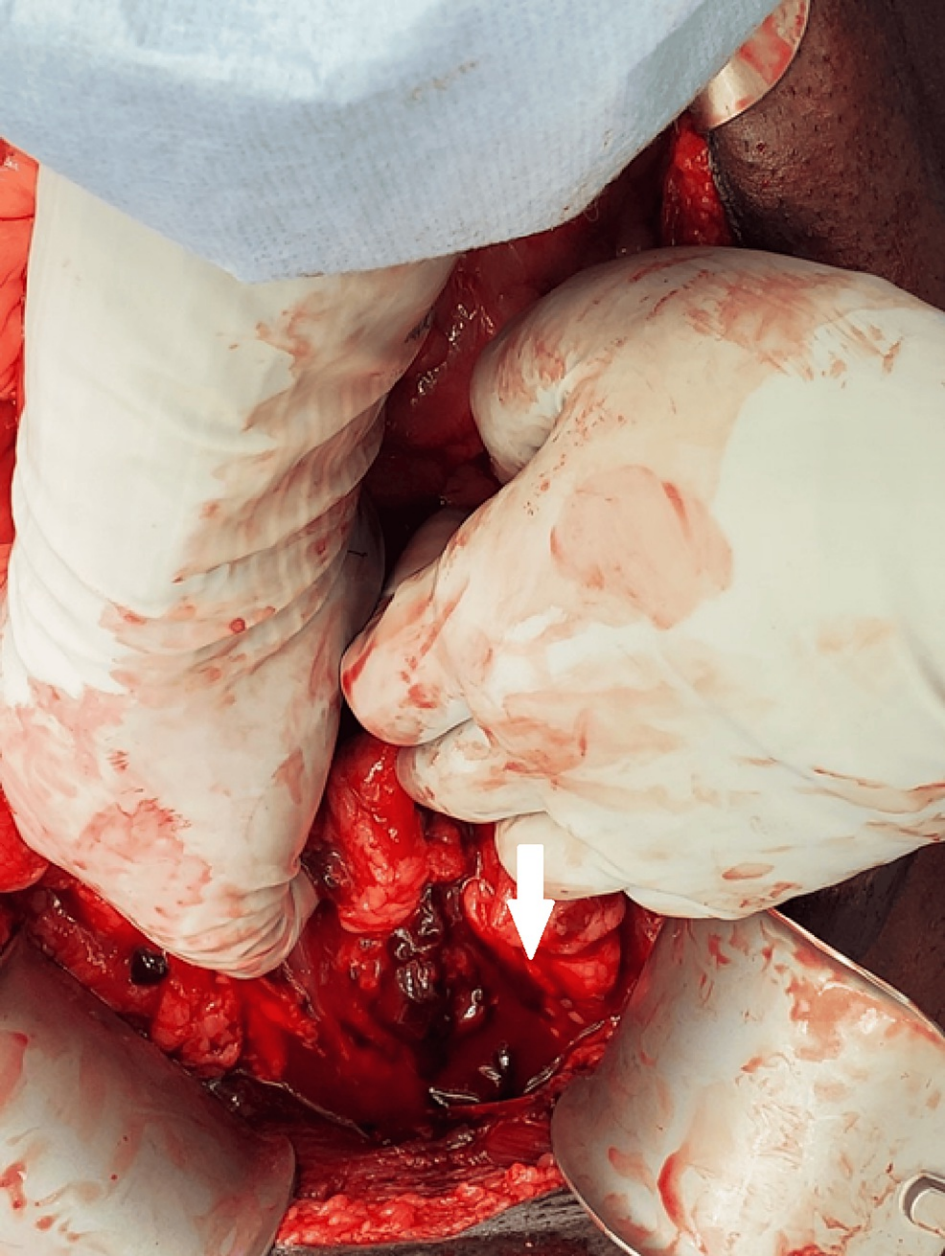
Injury and active hemorrhage in the space of Retzius and anterior bladder A bleeding contusion can be seen at the white arrow, which coincides with the anterior hole in the bladder.

**Figure 2:**
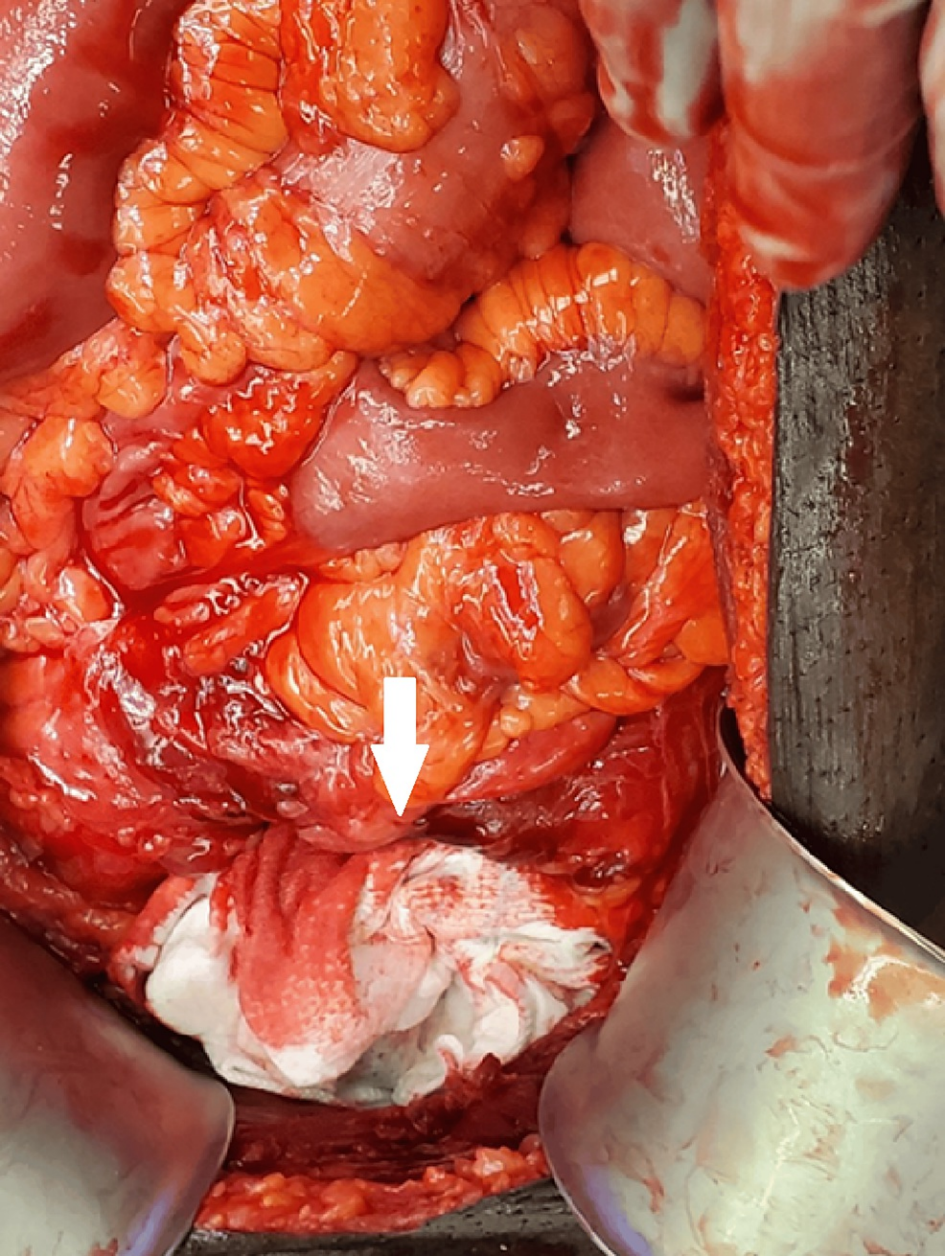
QuikClot Control+® 12x12 Hemostatic Device (C+) applied in the space of Retzius for tamponade and hemorrhage control The white arrow depicts a C+balled up around a laparotomy pad, which provides both an active hemostatic agent and tamponade via direct compression of the area. QuikClot Control+® 12x12 Hemostatic Device (C+): Teleflex Medical OEM, Plymouth, MN

A 43-year-old man presented to our urban, level-1 American College of Surgeons (ACS)-verified trauma institution after sustaining a gunshot wound to the right groin and left buttock. On arrival, his vitals were stable (BP: 190/85 mmHg; HR: 84 bpm; respirations: 18 bpm; temperature: 36.4 degrees C, and Glasgow Coma Scale (GCS): 15), but frank hematuria was noted. Contrast-enhanced computerized tomography (CT) revealed a bladder injury, an approximately 6x4 cm hematoma with active extravasation in the pelvis, and scattered hemo-pneumoperitoneum. He was immediately brought to the operating room for exploratory laparotomy. Upon entering the abdomen, approximately one liter of hemoperitoneum was identified, and a large, expanding Zone 3 hematoma was discovered to be larger than visualized on CT and decompressing into the peritoneal cavity. The bladder was mobilized to gain access into the space of Retzius. Evacuation of the hematoma revealed a raw wound bed with generalized oozing, active bleeding from numerous unnamed branch arteries and veins, and an additional hematoma surrounding the ballistic injury through the bladder (Figure 1). The space of Retzius was packed with C+ wrapped around a balled-up laparotomy pad for added volume (Figure 2).

This approach allowed the surgical team to gain hemorrhage control and quickly move to an examination of the abdomen. Here, we identified a through-and-through small bowel injury and two serosal tears, all of which were repaired primarily. There was minimal abdominal contamination. Roughly 10 minutes later, we returned our attention to the pelvis where the C+ had abated the bleeding from the retropubic space, and direct visualization of the surgical field was achievable. The bladder had an extensive anterior and posterior full thickness injury that was repaired in two layers. He had minimal further blood loss and required only one unit of packed red blood cells during the operation. A Foley catheter and peri-cystic drain were left in place. 

The patient did not require any further transfusions throughout his stay and was discharged within a week. At his two-week follow-up, the Foley catheter and drain were removed, drain creatinine was normal, and the CT cystogram was negative for a urine leak. The patient had an uneventful recovery with no acute kidney injury, bacteremia, or any other complications.

## Discussion

Penetrating injuries to the pelvis can be extremely challenging to manage given the narrow, highly vascular working space. Rapid hemostasis is critical to avoid further blood loss, more transfusions, trauma-induced coagulopathy, and ultimately morbidity and mortality [3]. Furthermore, failure to expeditiously identify and control a concomitant bowel injury can lead to serious late complications such as sepsis. Pre-peritoneal packing has been described for penetrating injuries to the pelvis but has traditionally been reserved for hemodynamically unstable patients [4]. While packing with laparotomy pads can provide tamponade, diffuse bleeding in complex injuries may be better managed with a combination of tamponade and an active hemostatic agent. Additionally, in the setting of increasing blood shortages, aggressive hemorrhage control and judicious transfusion of blood products make the use of hemostatic agents instrumental to patient care [2].

Previous versions of kaolin-impregnated gauze have been used as a packing adjunct during damage control laparotomies [4-6]. C+ applies kaolin more effectively than previous iterations and is rated for internal use. Currently, only animal models exist for the use of C+ in trauma, and in our literature review, only one other case study demonstrates the effect of C+ in a human trauma patient [4,7]. Given the complexity and location of the wound described in this case, we chose an aggressive management approach combining tamponade with an active hemostatic agent. Thus, similar to the approach described by Floan and Martin [7], we implemented C+ as an adjunct to our pre-peritoneal packing. In our patient, C+ assisted the operative team in quickly gaining hemorrhage control, minimizing blood loss, and limiting transfusions while the enterotomies were repaired and bowel spillage controlled.

Our case demonstrates the potential benefits of using a hemostatic dressing like C+ in the setting of large surface area bleeding. Leveraging C+ as a hemostatic adjunct allowed the operative team time to address other pressing injuries while hemostasis was achieved with the combination of tamponade and kaolin. Its use may have also decreased blood loss and the need for further transfusions in this patient. However, pre-peritoneal and peritoneal use of C+ has not been fully elucidated, and further robust investigations are required to determine the ultimate role of hemostatic dressings during trauma laparotomies.

## Conclusions

Obtaining swift hemorrhage control remains one of the fundamentals of trauma surgery. It is critically important to identify whether the source of bleeding requires sutures and to manage the non-surgical bleeding with available adjuncts. To our knowledge, this is only the second case report of the use of C+ in trauma. C+ may play a beneficial role in allowing the trauma surgeon to address bleeding from large surface area wounds, mitigate blood transfusions, obtain hemostasis, and attend more quickly to additional critical injuries. Additional inquiry may elucidate ideal circumstances in which to employ C+ for packing during trauma surgeries, such as a comparison of hemostasis induced in arterial versus venous bleeds, for instance. Future prospective studies on blood loss and transfusions are needed to further define its true benefit.
